# It’s time to shed some light on the importance of fungi in neonatal intensive care units: what do we know about the neonatal mycobiome?

**DOI:** 10.3389/fmicb.2024.1355418

**Published:** 2024-03-19

**Authors:** Dobrochna Wojciechowska, Sylwia Salamon, Katarzyna Wróblewska-Seniuk

**Affiliations:** ^1^II Department of Neonatology, Poznan University of Medical Sciences, Poznan, Poland; ^2^Doctoral School, Poznan University of Medical Sciences, Poznan, Poland; ^3^Department of Plant Microbiomics, Institute of Plant Genetics, Academy of Sciences, Poznan, Poland

**Keywords:** neonatal mycobiome, neonatal intensive care unit, microbial dysbiosis, fungal microbiome, vertical transmission, prematurity, DNA barcoding, next generation sequencing

## Abstract

The 21st century, thanks to the development of molecular methods, including DNA barcoding, using Sanger sequencing, and DNA metabarcoding, based on next-generation sequencing (NGS), is characterized by flourishing research on the human microbiome. Microbial dysbiosis is perceived as a new pathogenetic factor for neonatal diseases. Fungi are crucial, but neglected, components of the neonatal microbiome, which, despite their low abundance, significantly impact morbidity and mortality rates of premature infants hospitalized in Neonatal Intensive Care Units (NICUs). The neonatal mycobiome’s composition and effect on health remain poorly studied research areas. Our knowledge about neonatal mycobiome, composed of limited genera, is mainly based on research on the bacterial microbiome. We presume it is influenced by clinical factors, including prematurity, antibiotic therapy, and type of delivery. Understanding these risk factors may be useful in prevention strategies against dysbiosis and invasive fungal infections. Despite the methodological challenges resulting from the biology of the fungal cell, this topic is an attractive area of research that may contribute to more effective treatment, especially of newborns from risk groups. In this mini review, we discuss the current state of knowledge, research gaps, study difficulties, and future research directions on the neonatal mycobiome, concerning potential future clinical applications.

## Introduction

The twenty-first century has witnessed intensive microbiome research in both animal and human studies. Bacteria and fungi became essential elements of the ecosystem, instead of playing just the role of pathogens (Shan et al., 2019). The researchers have worked on various aspects concerning the microbiome’s development and function. It has become clear that the microbiome not only affects the infants’ health but it is an important modulator of the adults’ health in the long run. Microbial dysbiosis is correlated with abnormal development of the immune and central nervous systems, as well as problems with digestion and metabolism (Yao et al., 2021). The infant microbiome depends on both environmental and genetic factors. Prenatal transmission of the microbiome depends on the maternal (endometrial, vaginal, gastrointestinal, and oral) microbiota, which is affected by genetics, diet, medications (especially antibiotics), infections, and stress. Moreover, there are microbiome differences resulting from the mode of delivery. During vaginal delivery, the newborns acquire bacteria specific to maternal vagina, such as *Lactobacillus*, *Senathia* spp., and *Prevotella*, while during cesarean section (C-section), the newborns are colonized by maternal skin bacteria, including *Staphylococcus*, *Propionibacterium,* and *Corynebacterium* (Yao et al., 2021). Another critical factor for infant microbial dysbiosis is gestational age at birth. It is connected with interruption of foeatal development during the third trimester, statistically higher rates of C-sections, and the medicalization of early stages of life (Arboleya et al., 2022). Finally, neonatal microbiome depends on human milk administration and kangaroo mother care. Human milk microbiota is characterized by a low bacterial load of high diversity, with the most predominant taxa, *Staphylococcus* and *Streptococcus* (Boudry et al., 2021). Moreover, the microbiome of breast milk is subject to dynamic changes not only during lactation but also during a single feeding session (Laursen et al., 2021). Skin-to-skin contact is associated with improved health outcomes for both the infant and the mother. As we know from one of the latest studies, it also affects microbiota composition in early infancy and alters its development, as measured by volatility and microbiota age (Eckermann et al., 2024).

## Stop neglecting the fungi!

As mentioned earlier, most researchers have focused on the microbiome’s bacterial components while ignoring the role of fungi in microbiome studies. Such a situation may contribute to bias in the research process (Ward et al., 2017). As far as we know, fungi cover 13% of the adult gut microbiome and comprise over 100 genera (Schei et al., 2017). The human mycobiome participates in many physiological processes occurring in the intestines (e.g., nutrients and vitamins absorption, micronutrient biosynthesis) and plays an essential role in the immunological processes, such as the presentation of antigens via pattern recognition receptors (James et al., 2020; Paul et al., 2020). Animal studies prove that gut mycobiome depends on diet and age. Mycobiome dysbiosis is observed in common diseases, like inflammatory bowel disease, colorectal adenomas, pouchitis, and diarrhea, as well as in rare conditions such as graft versus host disease and Rett syndrome. The correlation between fungal dysbiosis/atopic diseases is often studied in the pediatric population (Glatthardt et al., 2023). Data published by Boutin’s research group revealed that a reduction of the relative abundance of the dominant age-associated fungal genus and/or an increase in fungal diversity in the first 3 months of life increases an infant’s risk of asthma at the age of 5 years (Boutin et al., 2021). However, it is still unclear whether fungal dysbiosis is a consequence of the disease or if it plays a role in its etiology (Huseyin et al., 2017). That is why there is still a need to study how the human mycobiome evolves from birth to death.

**Figure 1:**
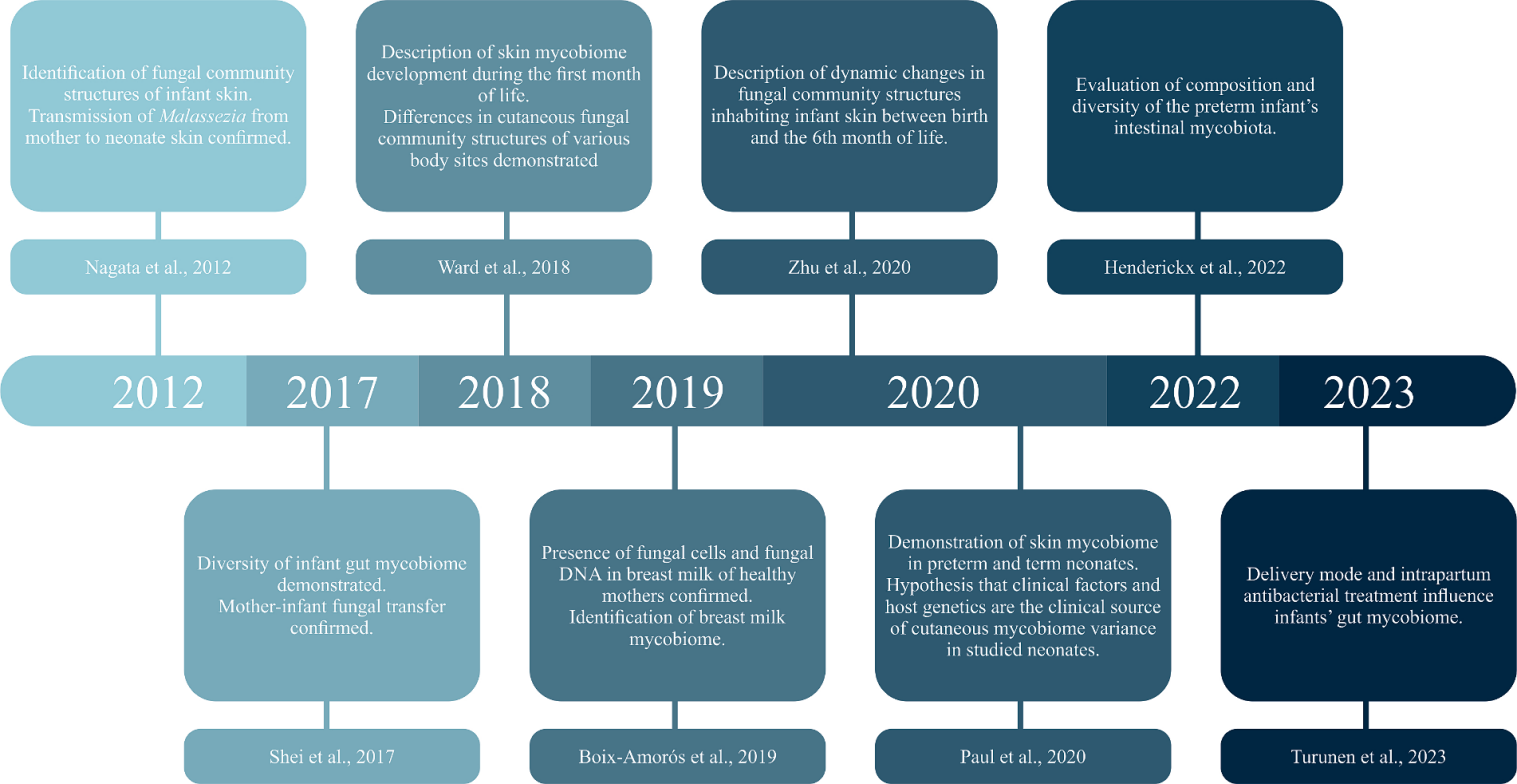
Timeline of selected milestones in mycobiome research.

The knowledge about pre- and postnatal transmission of the mycobiome is still limited; there are few studies on a similar subject. Figure 1 presents the timeline of selected milestones in mycobiome research. The first report, including a molecular analysis of the neonatal mycobiome, described 27 mother-infant pairs. The swabs were taken from the cheeks immediately after birth, then on the 1st and 3rd day of life, and 1 month after birth. The study confirmed vertical transmission of *Malassezia* (Nagata et al., 2012). Five years later, Schei et al. collected fecal samples from 298 mother-infant pairs. The maternal samples were collected at 35–38 gestational weeks of pregnancy and 3 months postpartum, while infant feces were gathered 10 days, 3 months, 1 year, and 2 years after birth. The mothers tended to have higher alpha and beta diversity in pregnancy than postpartum. The alpha diversity in the offspring seemed to increase steadily from birth, whereas the beta diversity was highest in 10-day-old infants. Interestingly, maternal fungal hosting made the offspring more susceptible to host fungi, which suggests that these mother-infant pairs share similar fungal hosting abilities. Because fungi are ubiquitous in the environment, they may originate from maternal genital tract and breastmilk, as well as contact with parental skin or hospital and home environment (Schei et al., 2017). As far as we know from the previous studies, breastmilk mycobiome is very diverse and consists of many fungal taxa (e.g., *Candida albicans, Candida parapsilosis, Cryptococcus neoformans, Saccharomyces cerevisiae, Candida glabrata*) differentiated mainly by geographic location and delivery mode (Boix-Amorós et al., 2019).

## New insights into the pathogenicity of fungi

On the other hand, fungi play a significant role as infectious agents, especially among preterm and very low birth weight (<1,500 g) infants hospitalized in the neonatal intensive care units (NICUs). Invasive fungal infections are a leading cause of morbidity and mortality in preterm neonates due to the risk factors such as immunodeficiency, immaturity of the natural protective barriers, and invasive procedures (e.g., intravenous access and parenteral nutrition, endotracheal intubation). *Candida* species are the most common cause of fungal infections in neonates and the third cause of late-onset sepsis in NICU patients, determining invasive infection with a high burden of morbidity (including late neurodevelopmental sequelae) and mortality (Barton et al., 2017; Bennett, 2017). Although *Candida albicans* has historically been the most frequently isolated species, recently non-albicans *Candida* (NAC) have emerged as important opportunistic pathogens (Juyal et al., 2013). For this reason, fluconazole prophylaxis should be considered in high-risk infants, especially those admitted to NICUs with high rates of invasive candidiasis (Kilpatrick et al., 2022). What is more, antibiotic therapy, frequently used in NICUs, causes an iatrogenic mycobiome dysbiosis, which leads to an overgrowth of species belonging to the pathobionts (e.g., *C. albicans, C. metapsilosis, C. parapsilosis,* and *C. tropicalis*) and an increased risk of fungal infections (James et al., 2020).

A great threat for hospitalized patients is the emergence of multi-drug resistant pathogens such as *Candida auris*, which may cause candidemia associated with high mortality rates (Forsberg et al., 2019). This fungal pathogen was initially identified in 2009 from the external ear canal of a 70-year-old Japanese woman (Satoh et al., 2009). In the following years, it has spread worldwide, causing significant diagnostic and treatment challenges. Conventional microbiology methods do not allow it to be identified. Furthermore, *C. auris* is characterized by distinct mechanisms of antifungal resistance to azoles and amphotericin B. The highest morbidity rates occur in adults, children and newborns requiring chronic treatment in intensive care units (Alvarado-Socarras et al., 2021). Based on the case series, the neonatal population mortality rate can be estimated from 37.5 to 80% (Chandramati et al., 2020; Alvarado-Socarras et al., 2021; Ramya et al., 2021).

## What is already known and current research gaps

Even though there are some previous studies concerning the neonatal mycobiome, they demonstrate mainly single-habitat descriptions. The best-studied area of research remains the intestine. The gut mycobiota was identified in most of the mothers and most of the offspring feces, supporting the thesis that fungi are an important element of the intestinal microbiome. The fungal species show a succession toward the maternal mycobiota as the child ages, with the most abundant *Debaryomyces hansenii* during breastfeeding and *Saccharomyces cerevisiae* after weaning (Schei et al., 2017; Turunen et al., 2023). The same issue was investigated by Henderickx et al., who showed that vaginally delivered infants present with the *Candida* genus. On the other hand, the *Malasseziomycetes* class and lower taxonomic levels are mainly characteristic for infants born through emergency C-section. Infants after vaginal and emergency C-section deliveries share fungi within the *Saccharomycetes* class but not for lower taxonomic levels. Infants delivered with a planned C-section are enriched in the *Microascales* and *Cladosporium* genera. Interestingly, while comparing newborns born prematurely with newborns born at term, *Candida* is the most abundant in both groups, but its load increases with gestational and postnatal age (Henderickx et al., 2022).

The second most frequently examined site is the skin. A previously mentioned study coordinated by Nagata revealed transmission of *Malassezia* spp. from mother to neonate (Nagata et al., 2012). Eight years later, a study on a larger group of children confirmed the dominance of the *Malassezia* genus and mainly *M.globosa* as the most abundant species. Skin sites and the subject’s age were the major factors accounting for the diversity and composition of skin mycobiomes (Zhu et al., 2020). Assuming that the immaturity of the newborn’s skin affects the skin mycobiome, a study on 15 full-term and 15 premature newborns was conducted. The most abundant genera in both preterm and term neonates were *Malassezia, Candida, Cladosporium, Fusarium*, and *Cryptococcus,* and the two most abundant species were *Malassezia restricta* and *Candida albicans.* Suprisingly no significant difference of relative abundances of these genera or species between preterm and term infants was found (Paul et al., 2020).

The study by Ward et al. shed a broader look at the newborn’s mycobiome. The swabs from the skin, oral cavity, and anus of the infants were collected over the first month of life. Moreover, the maternal anal and vaginal swabs were taken after labor. The early infant mycobiome consisted of a few taxa (e.g., *C. parapsilosis* and *C. tropicalis*), across all infant body sites. Unlike previously cited studies, *Malassezia* accounted for only 2% of the relative abundance of skin mycobiome. *C. parapsilosis, C. tropicalis, C. orthopsilosis*, and *S. cerevisiae* were the most abundant and prevalent species in infant oral mycobiomes during the first month of life. Among the anal samples, *C. parapsilosis, C. tropicalis, C. albicans*, and *S. cerevisiae* were most common. Similarly, the maternal mycobiomes (vaginal and anal) were also dominated by a single taxon, mainly *C. albicans*. Due to the small group size, the study did not show statistically significant differences between the mode of delivery and the composition of the neonatal mycobiome. Regarding the authors’ conclusions, there is a need to conduct further cross-sectional and longitudinal research with larger cohorts, allowing to gather a more complete mycobiome description (Ward et al., 2018).

Vertical transmission is considered one of the most important factors when analyzing the newborn’s mycobiome. Regarding research on the bacterial microbiome, studies on dyads (mother-infant pairs) make it possible to assess the impact of maternal health and mode of delivery on an infant’s microbiome. In the study by Heisel et al. breastmilk was obtained from mothers when infants were 1 month old, and the infants’ fecal samples were collected at 1 and 6 months of age. The most abundant and prevalent fungal species observed in breastmilk were *Paecilomyces dactylethromorphus*, *Fusarium equiseti, Malassezia restricta*, and *Candida albicans*. Similarly, the most abundant and prevalent fungal species in infant feces included *P. dactylethromorphus, M. restricta, C. albicans,* and *C. parapsilosis*. Moreover, bacterial-fungal correlations in breastmilk and infant feces were identified (Heisel et al., 2022). As far as we know, there is no data concerning kangaroo care, and its possible influence on neonatal mycobiome. Table 1 shows the most essential data about neonatal mycobiome and the gaps in knowledge that should be resolved in further studies. It emphasizes the need to conduct further research, considering not only mother-infant interactions but also associations regarding the non-fungal components of the microbiome.

**Table 1:** The most essential data about neonatal mycobiome and the gaps in knowledge.

What do we know?	Gaps in knowledge
Structure of fungal communities residing on infants’ skin (Nagata et al., 2012), in the gut (Schei et al., 2017), and in breast milk (Boix-Amorós et al., 2019).	Lack of functional studies of identified fungal communities. What is the role of identified fungi in healthy newborns? Knowledge about the entire mycobiome (altogether: skin, oral cavity, gut, breast milk) of mother-newborn pairs and its dynamic during the first few weeks after birth. Identification of beneficial fungal species and/or positive for human health fungal communities.
Fungi are transmitted from mother to offspring (Nagata et al., 2012; Shei et al., 2017).	Whether all genera of fungi associated with the mother can transfer to her offspring? Which pathway /and what type of transmission are predominant? Which factors can alter the transmission?
The infant mycobiome is altered by: delivery mode (Boix-Amorós et al., 2019; Paul et al., 2020; Heisel et al., 2022; Turunen et al., 2023), geographic location (Boix-Amorós et al., 2019), antibiotics exposure (Paul et al., 2020; Heisel et al., 2022; Turunen et al., 2023) host genetics – single nucleotide polymorphisms (SNPs) in genes mediating the response for fungal and bacterial expansion (Paul et al., 2020), diet (Paul et al., 2020), neonatal intensive care unit (NICU) environment (Paul et al., 2020).	Complete understanding of the impact of the environment on newborn mycobiome. How can the newborn mycobiome be modulated? Role of breastfeeding Kangaroo mother care What conditions favor the colonization of neonates by beneficial fungi? Do other genetic or epigenetic host components shape the mycobiome?
Correlation between bacterial and fungal taxa abundance in fecal and breast milk samples (Heisel et al., 2022).	Knowledge about the interaction and relationship between bacteria and fungi associated with neonate tissues and their role in health modulation.

## Methodologies and challenges in neonatal mycobiome studies

The era of molecular research has opened new opportunities to determine the human mycobiome. Molecular methods, including DNA barcoding using Sanger sequencing and DNA metabarcoding based on next-generation sequencing (NGS) methods, have replaced morphology-based identification due to more accurate and unambiguous results. The molecular approaches focus on DNA sequences (markers), which discriminate the fungal taxa. The official marker of fungal identification is the internal transcribed region (ITS), located in nuclear ribosomal RNA, which exhibits high genetic variations; thus, it is useful for fungal recognition (Raja et al., 2017). However, in the case of highly speciose genera of fungi (e.g., *Fusarium*, *Penicillium*, *Aspergillus*), the ITS sequence is insufficient to differentiate the species, so in those cases, additional DNA fragments are included in analyses (e.g., translation elongation factor, beta-tubulin, actin). DNA barcoding requires *in vitro* cultivation of fungal strains, which allows for detailed analyses to identify the fungal species; however, in this approach, the information about slow-growing and biotrophic fungi is lost.

On the contrary, DNA metabarcoding is culture-independent, so it can be performed directly from the tissue/body fluids. However, the identification with the use of most currently used NGS approach is limited to short ITS fragments (<500 base pair [bp]). To overcome the issue, we recommend using both DNA barcoding and metabarcoding or/and using the NGS Platform to obtain longer reads, providing higher taxonomic resolution (e.g., PacBio, Oxford Nanopore). Using this NGS platform, it is possible to obtain the sequences of the whole fungal rRNA operon of approximately 5,5 kbp in length (Lu et al., 2023).

The DNA extraction from fungal cells is challenging. Fungi, unlike bacteria, have a cell wall that is usually composed of, ß-1,3-glucan, ß-1,6-glucan, mannans, several glycoproteins and can also contain melanin or a rodlet layer (Thielemann et al., 2022). Thus, an essential step for fungal DNA extraction is efficient cell wall lysis by mechanic methods, like repeated beat-beating, followed by enzymatic cell lysis. Importantly, as with bacterial studies, standardization protocol for DNA isolation from fungal cells in human mycological studies is urgently needed. A sample-specific DNA extraction procedure with the possibility of its validation is recommended (Tiew et al., 2020). A wide range of ready-to-use techniques have been applied; for instance, the ZymoBIOMICS DNA Kit (Zymo Research, United States) was successfully used for human swab samples (Burton et al., 2022) and InviMag stool DNA kit (Stratec Molecular, Germany) preceded by mechanical and chemical cell lysis were adopted for breast milk samples (Boix-Amorós et al., 2019).

The molecular methods provide tremendous amounts of data; thus, the precise recruiting of the study groups is crucial for interpreting the obtained results. Considering that the human’s mycobiome composition can be affected by various factors, the specimen collection, sample processing, and the patient’s description must be prepared according to standardized procedures.

The most extensive and recommended database used for fungi taxonomy assignment is UNITE, based on data from the International Nucleotide Sequence Database Consortium (Nilsson et al., 2019). The UNITE contains 8,381,941 ITS sequences grouped into 223,659 fungal Species Hypotheses with digital object identifiers (DOIs) at a 1.5% threshold (as of 14 November 2023). The indicated database also contains non-fungal sequences that help distinguish fungi from other eukaryotes. Furthermore, it is constantly developed and allows for users’ data submission. However, it is essential to remember that the estimated number of fungal species is between 2.2 and 3.8 million, but much fewer are named and described (Hawksworth and Lücking, 2017). Thus, it might happen that the studied fungal community will not be entirely determined despite using large databases.

## Potential future developments in the field

The human microbiome is perceived as a primary target of future personalized medicine. Finding potential interactions between microbial dysbiosis and diseases may contribute to more effective treatment and disease prevention (Berg et al., 2020). Currently, the knowledge about the influence of probiotics on the neonatal mycobiome is poor. According to our knowledge the only study that assessed the impact of probiotics on the infants’ mycobiome is the previously mentioned research conducted by Schei et al. Study participants were enrolled into a population-based, randomized, placebo-controlled, and double-blinded trial on probiotics. The authors investigated how consuming milk with probiotic bacteria by pregnant and breastfeeding women affects the infants’ gut mycobiome. No statistically significant effects of maternal probiotic intake on offspring gut mycobiome were observed. Due to challenges in mycobiome research, the authors emphasize the need for further research (Schei et al., 2017, 2020).

Consequently, to the increasing rate of C-section, an interesting area of research remains vaginal seeding. During the procedure, a sterile gauze pad is placed in the mother’s vagina before the planned C-section. After delivery, the neonatal team wipes the baby’s mouth, face, and entire body with the gauze mentioned above. Scientists are investigating the effectiveness of this method in the transmission of the maternal reproductive tract flora to a newborn born by C-section. The first double-blind, randomized, placebo-controlled trial revealed that vaginal seeding significantly increases the bacterial load in the skin but not in the transitional stool and, when compared with the control group, causes a significant reduction in alpha diversity in the skin and transitional stool. The authors emphasize the need for further research to determine if these effects persist over time and whether they bring any health benefits (Mueller et al., 2023).

Another promising concept is maternal fecal microbiota transplantation. During the procedure, a processed maternal fecal sample, mixed with the mother’s milk (obtained before the C-section or, if needed, pasteurized bank milk), is administered in the first feeding of the infant within 2 h of birth. It has been shown that this procedure is more effective than vaginal seeding in the context of restoring the normal microbiome pattern in newborns born by C-section. This indicates a much more significant impact of fecal bacteria on the proper development of the newborn’s microbiome after birth (Korpela et al., 2020). Even though current research still lacks data to support this discovery in terms of mycobiome.

Considering differences resulting from gestational age and the occurrence of diseases typical for prematurely born newborns, like necrotizing enterocolitis (NEC), makes it possible to suspect the impact of mycobiome disorders on increased morbidity and mortality in this group of patients. Moreover, identifying the possible correlations between dysbiosis and complications related to premature birth may enable applying preventive strategies. NEC remains one of the most important and unresolved problems in neonatology, etiology of which is associated with intestinal dysbiosis. While no particular pathogen has been linked with NEC development, a general reduction in bacterial diversity and an increase in pathobiont abundance has been noted preceding disease onset. Currently, we do not have studies that would indicate the role of fungi in the development of NEC in newborns (Wilson et al., 2023). Research on mouse models suggests the potentially beneficial effects of β-glucan in preventing the development of NEC. B glucan is a kind of bioactive polysaccharide obtained from yeast, which, among many health-promoting activities, is also credited with a regulating effect on the microbiome. Despite promising results indicating the effect of β-glucan administration on intestinal injury of NEC mice, further research is needed to determine whether a similar phenomenon is observed in newborns (Zhang et al., 2023).

## Conclusion

The neonatal mycobiome remains neglected despite intensive research on the human microbiome. Molecular methods open up new research possibilities, which may bring us closer to personalized medicine in this susceptible group of patients, especially those born prematurely. Analysis of current studies leaves many research gaps and opportunities for potential future developments, such as the impact of vertical transmission on the development of the infant’s mycobiome and the role of probiotics and microbiota transplant in restoring microbial balance. Moreover, it is crucial to clarify the role of fungi as potential factors in the etipathogenesis of prematurity-related diseases, such as necrotizing enterocolitis.

To conclude, it is recommended that further cross-sectional studies on larger cohorts, incorporating the maternal mycobiome status, should be conducted.

## Author contributions

DW: Conceptualization, Investigation, Visualisation, Writing – original draft. SS: Methodology, Writing – original draft. KW-S: Writing – review & editing.
